# Lung cancer screening health beliefs among a high-risk population in China: a cross-sectional survey

**DOI:** 10.3389/fpubh.2026.1759430

**Published:** 2026-10-02

**Authors:** Xiujing Lin, Fangfang Wang, Yiyang Lin, Jiani Yang, Fang Lei, Feifei Huang

**Affiliations:** 1 School of Nursing, Fujian Medical University, Fuzhou, Fujian, China; 2 Peking Union Medical College Hospital, Peking Union Medical College, Chinese Academy of Medical Science, Beijing, China; 3 School of Nursing, University of Minnesota, Twin Cities, Minneapolis, MN, United States

**Keywords:** China, health belief model, health beliefs, high-risk population, lung cancer, lung cancer screening

## Abstract

**Objectives:**

Evidence on lung cancer screening (LCS) health beliefs among high-risk populations in China remains limited. This study aimed to examine LCS health beliefs and associated factors among individuals at high risk for lung cancer in Fujian Province, China.

**Methods:**

This cross-sectional study was conducted from September 2020 to October 2022. High-risk individuals were recruited using a four-stage multistage sampling procedure incorporating probability-proportionate-to-size sampling. LCS health beliefs were assessed using the Chinese Lung Cancer Screening Health Belief Scale (LCSHB-C). Multiple linear regression was used to examine associated factors.

**Results:**

A total of 1,957 participants were included, with a mean overall LCSHB-C score of 95.10 (SD = 10.13). LCS-related knowledge and previous screening experience showed the most consistent positive associations with health beliefs. Rural residence, religious affiliation, and higher household income were also associated with higher overall scores, whereas female sex, current or former smoking, COPD, and participation in the national basic medical insurance program were associated with lower scores. Associations with age, education, body mass index, and occupational exposure varied across health belief domains.

**Conclusion:**

LCS health beliefs were associated with screening knowledge, previous screening experience, and several sociodemographic and health-related characteristics. These findings may inform tailored LCS education and support strategies to facilitate informed participation among high-risk populations.

## 1 Introduction

Lung cancer is highly prevalent and remains the leading cause of cancer-related deaths worldwide (1). China bears a substantial burden of lung cancer, with approximately 1.18 million new cases reported in 2024, accounting for 22.8% of all cancer cases in China. Lung cancer also accounted for approximately 0.74 million deaths in China in 2024, representing 28.8% of all cancer deaths (2). The high mortality burden of lung cancer underscores its poor prognosis and substantial public health impact. Studies have consistently shown that the 5-year survival rate is closely linked to the stage at diagnosis (3), underscoring the critical importance of early detection and treatment for improving lung cancer outcomes (4).

Low-dose computed tomography (LDCT) has been widely recommended as the preferred screening test for lung cancer due to its high sensitivity and reasonable specificity, according to most clinical practice guidelines (3, 5–7). The Chinese Guideline for Lung Cancer Screening and Early Diagnosis and Treatment identifies individuals aged 50–74 years with risk factors, including a history of smoking, long-term passive smoking exposure (including second-hand smoke and cooking-oil fumes), chronic obstructive pulmonary disease, occupational exposures, or a family history of lung cancer, as a high-risk population for lung cancer and recommends regular screening with LDCT (3). However, despite its demonstrated benefits, LDCT screening uptake remains suboptimal, with only 1.7–35% of eligible individuals worldwide reported to have ever undergone LDCT screening (8–13). This issue is particularly prominent in China, where screening rates among high-risk populations have been reported to range from 6 to 35% (9, 10, 14). Therefore, a better understanding of the factors associated with lung cancer screening (LCS) participation is important for informing strategies to promote appropriate screening among high-risk populations.

The Health Belief Model (HBM) is a social cognition framework widely used to understand health-related behaviors, including participation in disease prevention and screening programs (15). It encompasses key concepts, including perceived benefit, perceived barriers, perceived risk, and self-efficacy. Perceived benefit refers to an individual’s belief in the potential advantages of engaging in a health behavior, while perceived barriers involve the psychological and tangible costs or obstacles associated with taking such action. Perceived risk reflects an individual’s perception of the likelihood or threat of developing a disease, and self-efficacy reflects the individual’s confidence in their ability to take action. In the present study, LCS health beliefs are operationalized through these specific, validated multidimensional constructs of the HBM rather than as a general attitudinal concept. Understanding these constructs may help healthcare providers identify potentially relevant perceptions and barriers associated with LCS and inform the development of future screening promotion strategies.

Theory-informed approaches are important for the development of health-promotion interventions (16). The HBM is one such framework that has consistently been identified as relevant to cancer screening behaviors across various studies (17, 18). For example, research has shown that breast cancer screening participation is associated with greater perceived risk and perceived benefits and fewer perceived barriers. These constructs, together with intrapersonal characteristics such as age, gender, and health literacy and interpersonal or contextual characteristics such as family history and income, have also been associated with cancer screening behaviors (16, 19).

Previous studies suggest that breast cancer screening interventions tailored to relevant health-belief constructs may improve mammography uptake (16, 19). However, LCS differs from breast and colorectal cancer screening in several ways. LCS eligibility is strongly related to smoking history, and individuals eligible for LCS may therefore encounter distinctive psychosocial considerations. For instance, long-term smokers may experience cancer-related stigma and mistrust that can shape perceptions of lung cancer and screening (20, 21). Additionally, the perceived self-inflicted risks associated with smoking may introduce psychosocial complexities that are particularly relevant to LCS decision-making (22, 23). Cultural context may also be associated with health beliefs and screening perceptions (24, 25). Previous research has suggested that cultural beliefs concerning fate, personal control, and illness may shape perceptions of cancer prevention. However, these relationships may vary across populations and contexts (26). Given these potentially relevant contextual factors, it is important to examine LCS health beliefs within specific populations and to explore how sociodemographic and health-related characteristics are associated with different HBM constructs.

However, existing studies have primarily focused on decision-making regarding LCS or on actual screening behaviors and their associated factors (24, 27). Although health beliefs capture individuals’ perceptions of lung cancer risk, screening benefits and barriers, and confidence in undertaking screening-related actions, and have been associated with screening behaviors, research specifically addressing health beliefs in the context of LCS remains relatively limited. In addition, previous studies have often involved relatively small samples or used assessment instruments with limited psychometric validation (20, 21). Some studies have used qualitative approaches to provide in-depth insights into screening perceptions and experiences (22, 24, 25, 28), whereas others have included participants with varying levels of lung cancer risk, potentially limiting their applicability to populations specifically eligible for LCS. Furthermore, the associations between the core HBM constructs and modifying characteristics, including sociodemographic and health-related factors, remain insufficiently understood (29–31).

Considering the predicted substantial increase in lung cancer cases over the next decade (32), promoting informed participation in LCS among high-risk populations remains an important public health priority. To better understand LCS-related perceptions among individuals at elevated risk, the present study focused on individuals at high risk for lung cancer recruited from two cities in Fujian Province, China. Specifically, we systematically assessed their LCS health beliefs and examined the associations of sociodemographic and health-related characteristics with overall LCS health beliefs and the core HBM constructs. It is also important to note that this study was conducted during the coronavirus disease (COVID-19) pandemic. The pandemic may have influenced both public perceptions of respiratory health and access to routine healthcare services, including cancer screening (33, 34). Accordingly, the findings should be interpreted within this specific temporal and healthcare context. The present study aimed to provide empirical evidence on LCS-related health beliefs and their associated factors among individuals at high risk for lung cancer in Fujian Province, China, and to inform the development of future strategies aimed at promoting informed participation in LCS.

## 2 Materials and methods

### 2.1 Design

A large-scale cross-sectional study was conducted among adults at high risk for lung cancer in urban and rural areas of Fuzhou and Putian, Fujian Province, China, from September 2020 to October 2022. The study was reported in accordance with the Strengthening the Reporting of Observational Studies in Epidemiology (STROBE) statement.

### 2.2 Measures

The study questionnaire consisted of two parts: sociodemographic and health-related characteristics and LCS health beliefs. The Chinese version of the Lung Cancer Screening Health Belief Scales (LCSHB-C) was used to assess LCS-related health beliefs among individuals at high risk for lung cancer (35). The instrument comprised four subscales: perceived risk of lung cancer (3 items), perceived benefits of LCS (6 items), perceived barriers to LCS (17 items), and self-efficacy for LCS (9 items), with each item rated on a four-point Likert-type scale. Subscale scores were calculated by summing the corresponding item scores, and an overall LCS health belief score was calculated according to the scoring procedure of the LCSHB-C.

Higher scores indicated greater perceived risk of lung cancer, greater perceived benefits of LCS, and greater self-efficacy for LCS. For the perceived-barriers subscale, the scoring direction was reversed. Therefore, a higher score indicated fewer perceived barriers, whereas a lower score indicated greater perceived barriers to LCS. Thus, the numerical score of the perceived barriers subscale should be distinguished from the underlying level of perceived barriers when interpreting the findings. The Cronbach’s alpha coefficients for the four subscales ranged from 0.88 to 0.92, and the content validity indices ranged from 0.87 to 1.00 (35).

Sociodemographic and health-related variables were selected a priori based on previous literature and the HBM framework and included age, sex, residential area, educational level, occupation, per capita monthly household income, family history of cancer, body mass index (BMI), type of medical insurance, history of chronic obstructive pulmonary disease (COPD), smoking status, religious affiliation, history of LCS, and history of occupational exposure. Additionally, two questions assessing knowledge of the recommended LCS method and screening frequency were included.

### 2.3 Study setting and participants

Participants were recruited from Fuzhou and Putian, Fujian Province, southeastern China, using a four-stage multistage sampling procedure incorporating probability-proportionate-to-size (PPS) sampling. Sampling was conducted separately in urban and rural areas. The sampling and recruitment process is shown in Figure 1.

**Figure 1:**
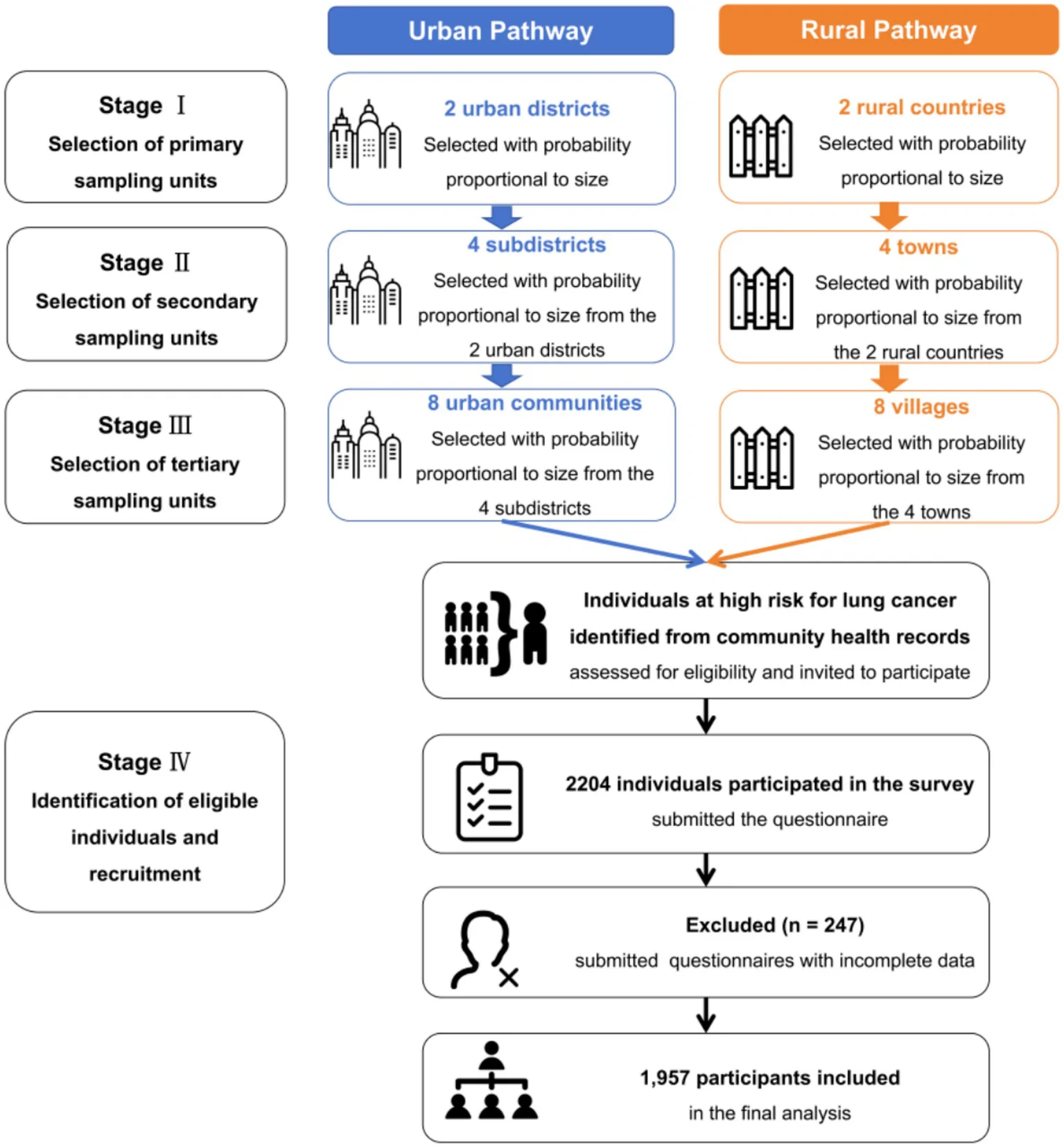
The five-stage probability proportionate to size sampling process used in the study.

Participants were eligible if they met the criteria for a population at high risk for lung cancer according to the Chinese guideline for lung cancer screening and early detection (3): (1) aged 50–74 years; (2) had at least one of the following risk factors: a smoking history of at least 30 pack-years and currently smoking or having quit within the previous 15 years; prolonged exposure to passive smoking (living or working with smokers for ≥20 years); a history of COPD; occupational exposure to asbestos, radon, beryllium, chromium, cadmium, nickel, silicon, soot, or coal soot for at least 1 year; or a family history of lung cancer; and (3) were able to understand and complete the study questionnaire. Individuals with a previous diagnosis of lung cancer, mental disorders, or cognitive impairment that prevented completion of the questionnaire were excluded. Relevant health information used to determine eligibility was obtained from community health records.

The minimum required sample size was estimated using the formula $\frac{4U\alpha^{2}S^{2}}{\delta^{2}}$ , where *S* represented the standard deviation of the LCS health belief score among individuals at high risk for lung cancer (*S* = 9.26), *U*α = 1.96, and *δ* = 0.25S (36). The calculation yielded a minimum sample size of 246. After allowing for an anticipated 20% nonresponse or invalid-response rate, the minimum target sample size was 295 participants. Because the study used a multistage population-based sampling procedure across the selected study sites, recruitment continued across all sampled units, resulting in a final analytic sample larger than the minimum required sample size.

### 2.4 Data collection

Data were collected by trained researchers using standardized procedures. Before questionnaire administration, eligible participants were informed of the study purpose, procedures, voluntary nature of participation, and confidentiality of their responses. After providing written informed consent, participants completed the electronic questionnaire via Wenjuanxing,^1^ with assistance from trained researchers when needed. Completed questionnaires were checked for completeness and logical consistency. Questionnaires with incomplete responses or predefined logical inconsistencies were excluded from the analysis. The final analyses were therefore based on complete valid questionnaires, and no missing-data imputation was performed.

### 2.5 Data analysis

Statistical analyses were performed using SPSS version 26.0. Continuous variables were summarized as means and standard deviations (SDs), and categorical variables as frequencies and percentages. Multiple linear regression analyses were conducted to examine factors associated with LCS health belief scores. Candidate independent variables were selected a priori based on previous literature and the HBM and were entered simultaneously into the regression models. Categorical variables were dummy coded, with reference categories specified in Appendix 1.

Multicollinearity among independent variables was assessed using variance inflation factors (VIFs) and tolerance values. Residual plots were examined to assess the distribution of residuals and potential deviations from linearity and homoscedasticity. Given departures from ideal residual patterns in some models, sensitivity analyses were conducted using heteroskedasticity-robust (HC3) standard errors. Model fit was evaluated using *R*^2^ and adjusted *R*^2^. Regression results are reported as unstandardized regression coefficients (*B*) with 95% confidence intervals (CIs). All statistical tests were two-sided, with *p* < 0.05 considered statistically significant. No formal adjustment for multiple testing was applied. The regression analyses were conducted at the individual level without incorporating sampling weights or adjustments for the complex sampling design.

## 3 Results

### 3.1 Participant characteristics

Of the 2,204 eligible individuals who agreed to participate, 1957 completed the questionnaire and were included in the final analysis, yielding a completion rate of 88.79%. The remaining 247 individuals submitted incomplete questionnaires and were excluded from the analysis. The mean age of the participants was 61.54 years (SD = 8.93). Participant characteristics are presented in Table 1.

**Table 1:** Average of LCS health belief level by descriptive characteristic of participants (*N* = 1957).

Variable	Total *n* (%)	Perceived barrier	Perceived benefit	Perceived risk	Self-efficacy	Total score of health belief
Gender						
Male	1,162 (59.4%)	44.54 ± 7.28	18.10 ± 2.87	5.75 ± 2.13	26.62 ± 5.17	95.01 ± 10.54
Female	795 (40.6%)	45.92 ± 5.83	18.04 ± 2.50	5.30 ± 1.86	25.98 ± 4.84	95.24 ± 9.51
Religion						
No	690 (35.3%)	44.84 ± 6.97	18.35 ± 2.93	5.71 ± 1.97	26.75 ± 4.93	95.65 ± 10.29
Yes	1,267 (64.7%)	45.24 ± 6.64	17.93 ± 2.59	5.49 ± 2.06	26.15 ± 5.11	94.81 ± 10.03
Residence area						
Urban	910 (46.5%)	44.20 ± 6.71	18.14 ± 2.79	5.75 ± 2.01	26.34 ± 5.15	94.44 ± 9.99
Rural	1,047 (53.5%)	45.88 ± 6.71	18.02 ± 2.67	5.41 ± 2.04	26.37 ± 4.96	95.68 ± 10.22
Educational level						
Primary school degree or below	709 (36.2%)	45.42 ± 6.18	17.74 ± 2.56	5.26 ± 1.96	25.36 ± 5.18	93.77 ± 9.97
Junior high school degree	603 (30.8%)	44.96 ± 6.88	18.12 ± 2.66	5.58 ± 2.05	26.48 ± 4.83	95.14 ± 9.64
Senior high school degree	496 (25.3%)	45.25 ± 6.45	18.35 ± 2.58	5.81 ± 1.99	27.16 ± 4.53	96.57 ± 9.41
Bachelor’s degree or higher	149 (7.6%)	43.63 ± 9.33	18.58 ± 3.83	6.19 ± 2.18	28.01 ± 5.90	96.41 ± 13.76
Type of medical insurance						
Self-pay	75(3.8%)	43.69 ± 11.05	17.89 ± 4.55	6.81 ± 2.70	27.69 ± 6.86	96.09 ± 16.78
NBMI for urban employed	715 (36.5%)	45.69 ± 5.93	18.22 ± 2.60	5.66 ± 1.82	26.95 ± 4.43	96.51 ± 9.28
NBMI for urban and rural residents	1,167 (59.6%)	44.83 ± 6.86	18.01 ± 2.64	5.43 ± 2.08	25.91 ± 5.22	94.18 ± 9.97
Smoking status						
No smoking	874 (44.7%)	46.66 ± 4.99	18.07 ± 2.34	5.21 ± 1.71	26.10 ± 4.53	96.04 ± 8.56
Currently smoking	827 (42.3%)	43.61 ± 7.89	18.08 ± 3.03	5.80 ± 2.25	26.57 ± 5.52	94.06 ± 10.85
Quit smoking	256 (13.1%)	44.59 ± 6.95	18.09 ± 2.90	6.04 ± 2.11	26.58 ± 5.13	95.29 ± 12.18
Family history of cancer						
No	1797 (91.8%)	45.27 ± 6.68	18.10 ± 2.69	5.48 ± 1.99	26.40 ± 5.05	95.25 ± 10.11
Unclear	116 (5.9%)	42.16 ± 7.14	17.76 ± 3.06	6.78 ± 2.36	25.57 ± 4.65	92.28 ± 8.95
Yes	44 (2.2%)	45.91 ± 7.24	18.00 ± 3.21	6.05 ± 1.57	26.75 ± 6.08	96.70 ± 12.69
Considered LDCT as lung cancer screening method						
No	1,229 (62.8%)	43.98 ± 6.61	17.65 ± 2.69	5.54 ± 2.05	25.48 ± 5.02	92.65 ± 9.16
Yes	728 (37.2%)	46.98 ± 6.59	18.80 ± 2.63	5.62 ± 2.01	27.84 ± 4.74	99.25 ± 10.34
LDCT should be conducted once a year						
No	1,052 (53.8%)	43.51 ± 6.99	17.68 ± 2.81	5.56 ± 2.17	25.33 ± 5.32	92.09 ± 9.78
Yes	905 (46.2%)	46.94 ± 5.98	18.54 ± 2.55	5.57 ± 1.86	27.56 ± 4.43	98.61 ± 9.38
Occupation						
Worker	907 (46.3%)	44.58 ± 7.24	17.91 ± 2.79	5.46 ± 2.20	25.87 ± 5.35	93.83 ± 10.23
Civil servant	117 (6.0%)	42.39 ± 8.63	17.85 ± 3.85	6.91 ± 2.27	27.50 ± 5.92	94.65 ± 14.64
Businessperson	205 (10.5%)	42.62 ± 7.56	18.14 ± 3.13	6.08 ± 2.15	27.43 ± 5.58	94.27 ± 11.36
Retired/unemployed	728 (37.2%)	46.88 ± 4.84	18.30 ± 2.25	5.34 ± 1.59	26.48 ± 4.22	97.00 ± 8.33
History of occupational exposure						
No	1,484 (75.8%)	45.40 ± 6.44	18.11 ± 2.54	5.38 ± 1.95	26.32 ± 5.05	95.21 ± 9.43
Yes	473 (24.2%)	44.15 ± 7.63	17.97 ± 3.23	6.15 ± 2.18	26.50 ± 5.06	94.77 ± 12.08
History of lung cancer screening						
No	1,115 (57.0%)	43.84 ± 6.65	17.63 ± 2.69	5.40 ± 2.08	25.23 ± 5.03	92.09 ± 9.32
Yes	842 (43.0%)	46.76 ± 6.56	18.67 ± 2.66	5.80 ± 1.94	27.86 ± 4.68	99.09 ± 9.78
Per capita monthly household income (USD)						
<$143	164(8.4%)	45.17 ± 7.44	17.79 ± 3.12	5.04 ± 1.93	25.16 ± 5.79	93.16 ± 12.22
$144 ~ $428	786 (40.2%)	44.85 ± 6.00	18.01 ± 2.49	5.33 ± 2.05	25.64 ± 5.02	93.84 ± 9.56
$429 ~ $713	673 (34.4%)	45.49 ± 6.98	18.11 ± 2.53	5.79 ± 2.00	26.62 ± 4.49	96.02 ± 9.08
≥$714	334 (17.1%)	44.84 ± 7.60	18.30 ± 3.34	5.93 ± 1.96	28.12 ± 5.32	97.19 ± 11.66

COPD, chronic obstructive pulmonary disease; LDCT, low-dose CT; LCS, lung cancer screening; NBMI, national basic medical insurance program.

### 3.2 Descriptive statistics of the LCSHB-C

The mean total LCSHB-C score was 95.10 (SD = 10.13). Descriptive statistics for the total and subscale scores are presented in Table 2. Table 3 presents the five highest- and five lowest-scoring LCSHB-C items.

**Table 2:** Lung Cancer Screening Health Belief Scale score ranges by dimension.

Dimension	Mean	SD	Score range	Number of items	Standardized score
Perceived barrier	45.10	6.76	17–68	17	66.32
Perceived benefit	18.08	2.72	6–24	6	75.33
Perceived risk	5.57	2.03	3–12	3	46.42
Self-efficacy	26.36	5.05	9–36	9	73.22
Total	95.10	10.13	35–140	35	67.93

Standard score = (mean of scale or dimensions ÷ full score of each entry) × 100.

**Table 3:** The five lowest and highest scored lung cancer screening health belief items by rank.

Item	Mean	Standard deviation	Rank
Benefit 1: Having a lung scan will help find lung cancer early.	3.04	0.51	1
Benefit 5: Having a lung scan will help my family not worry as much.	3.02	0.54	2
Benefit 6: Having a lung scan will give me peace of mind.	3.02	0.55	3
Benefit 3: Having a lung scan will help me not worry as much about lung cancer.	3.00	0.53	4
Self-efficacy 3: How confident are you that you can find transportation to get to and from the clinic/hospital to have a lung scan?	3.00	0.68	5
Barrier 4: I might put off having a lung scan because no one in my family had lung cancer.	2.36	0.68	31
Barrier 6: I might put off having a lung scan because I do not have any lung problems or symptoms.	2.29	0.63	32
Risk 1: It is likely that I will get lung cancer sometime in my lifetime.	1.94	0.76	33
Risk 2: It is likely that I will get lung cancer in the next 10 years.	1.83	0.72	34
Risk 3: It is likely that I will get lung cancer in the next 5 years.	1.81	0.71	35

### 3.3 Factors associated with LCS health beliefs

Multiple linear regression analyses were conducted to examine factors associated with the overall and domain-specific LCS health belief scores. All five regression models were statistically significant (all *p* < 0.001), explaining 9.6–31.2% of the variance in the overall and domain-specific scores. The model for the overall LCSHB-C score explained 31.2% of the variance (*R*^2^ = 0.312; adjusted *R*^2^ = 0.303; *F* = 33.648, *p* < 0.001).

For the overall LCSHB-C score, higher scores were associated with rural residence (*B* = 3.276, 95% CI: 2.353 to 4.199), correctly identifying LDCT as an LCS method (*B* = 4.912, 95% CI: 4.106 to 5.719), recognizing annual LDCT screening (*B* = 4.640, 95% CI: 3.854–5.425), and a history of LCS (*B* = 5.717, 95% CI: 4.885 to 6.548). Religious affiliation and higher household income were also positively associated with the overall score (*B* = 0.917, 95% CI: 0.053 to 1.781). In contrast, a history of COPD (*B* = −4.820, 95% CI: −6.275 to −3.365), female sex (*B* = −1.627, 95% CI: −2.856 to −0.398), and current (*B* = −1.722, 95% CI: −3.093 to −0.351) or former smoking (*B* = −1.802, 95% CI: −3.363 to −0.241) were associated with lower overall scores. Participation in the national basic medical insurance program was also negatively associated with the overall score.

Associations varied across the four health belief domains. LCS-related knowledge and previous screening experience showed the most consistent patterns. Correctly identifying LDCT as the screening method and recognizing annual screening were positively associated with perceived benefits, fewer perceived barriers, and self-efficacy, while a history of LCS was positively associated with all four domain scores. Rural residence was associated with fewer perceived barriers and greater self-efficacy. Higher educational attainment was primarily associated with greater perceived benefits and self-efficacy, whereas higher household income was more consistently associated with perceived risk and self-efficacy.

Several health-related characteristics showed different patterns across domains. Older age was associated with fewer perceived barriers (*B* = 0.127, 95% CI: 0.089 to 0.164) but lower perceived risk (*B* = −0.021, 95% CI: −0.033 to −0.009). A history of COPD was associated with greater perceived risk (*B* = 0.620, 95% CI: 0.290 to 0.950), but with greater perceived barriers (*B* = −4.187, 95% CI: −5.186 to −3.188) and lower self-efficacy (*B* = −1.199, 95% CI: −1.991 to −0.406). Occupational exposure showed a similar domain-specific pattern, being associated with greater perceived risk but lower perceived benefits and self-efficacy. Higher scores on the perceived-barriers subscale indicate fewer perceived barriers to LCS.

Multicollinearity diagnostics indicated that most VIF values were below 5, although higher values were observed for two medical insurance categories (VIFs = 7.202 and 7.753), no VIF exceeded 10. Residual diagnostics indicated some departures from ideal residual patterns in several models. Accordingly, sensitivity analyses using HC3 robust standard errors were conducted and yielded findings generally consistent with the primary analyses, with no material changes in the direction or overall pattern of the principal associations (Appendices 2 and 3). Complete regression estimates, including unstandardized regression coefficients (*B*), 95% CIs, *p* values, and reference categories, are presented in Figures 2–6, Appendix 1.

**Figure 2:**
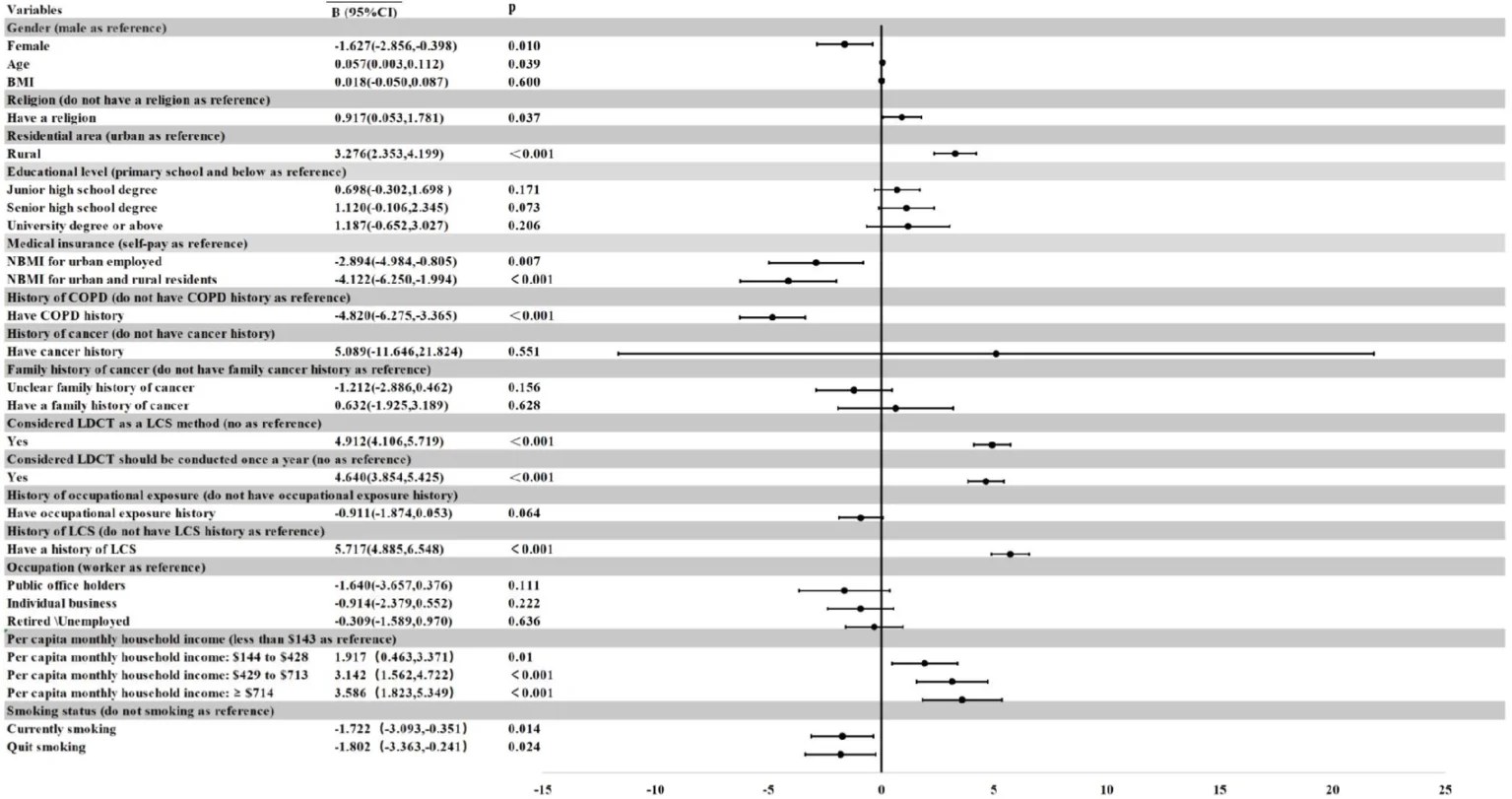
Forest plot showing the association between lung cancer health beliefs and associated factors. *R^2^* = 0.312, adjusted *R^2^* = 0.303, *F* = 33.648, *p* < 0.001, the B reported is unstandardized regression coefficients.

**Figure 3:**
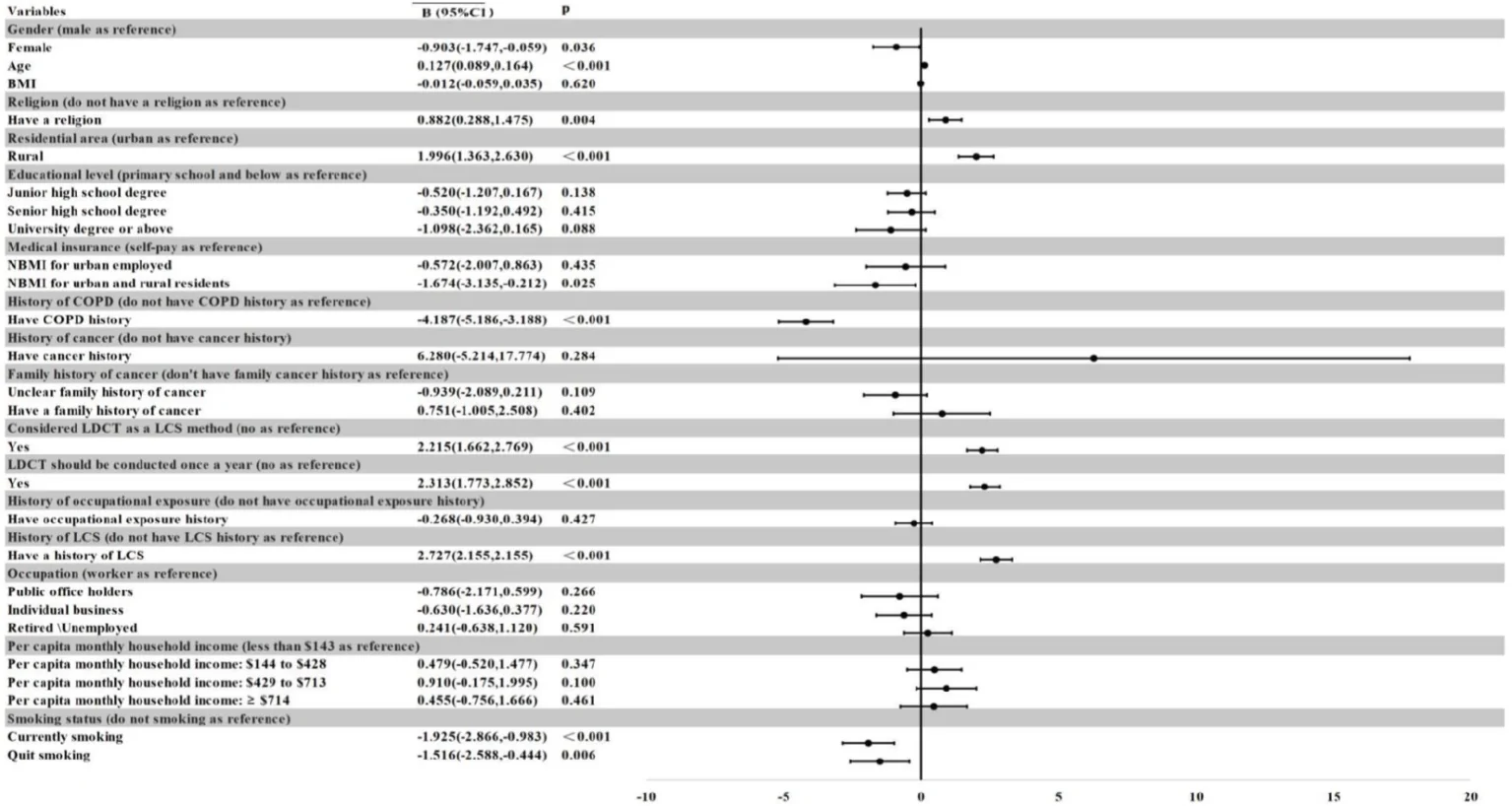
Forest plot showing the association between perceived barriers of LCS and associated factors. *R^2^* = 0.272, adjusted *R^2^* = 0.262, *F* = 27.692, *p* < 0.001, the B reported is unstandardized regression coefficients.

**Figure 4:**
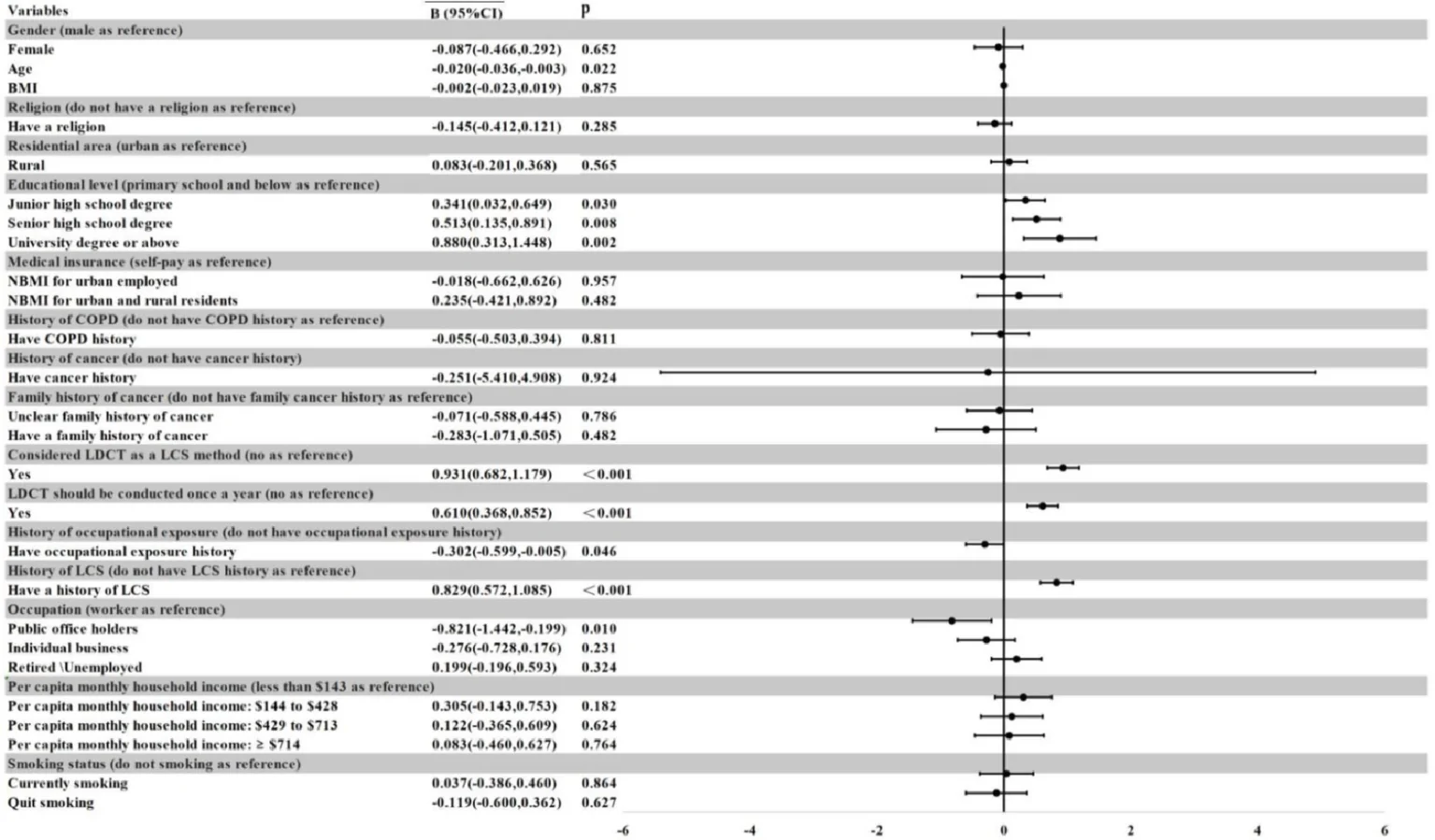
Forest plot showing the association between perceived benefits of LCS and associated factors. *R^2^* = 0.096, adjusted *R^2^* = 0.084, *F* = 7.877, *p* < 0.001, the B reported is unstandardized regression coefficients.

**Figure 5:**
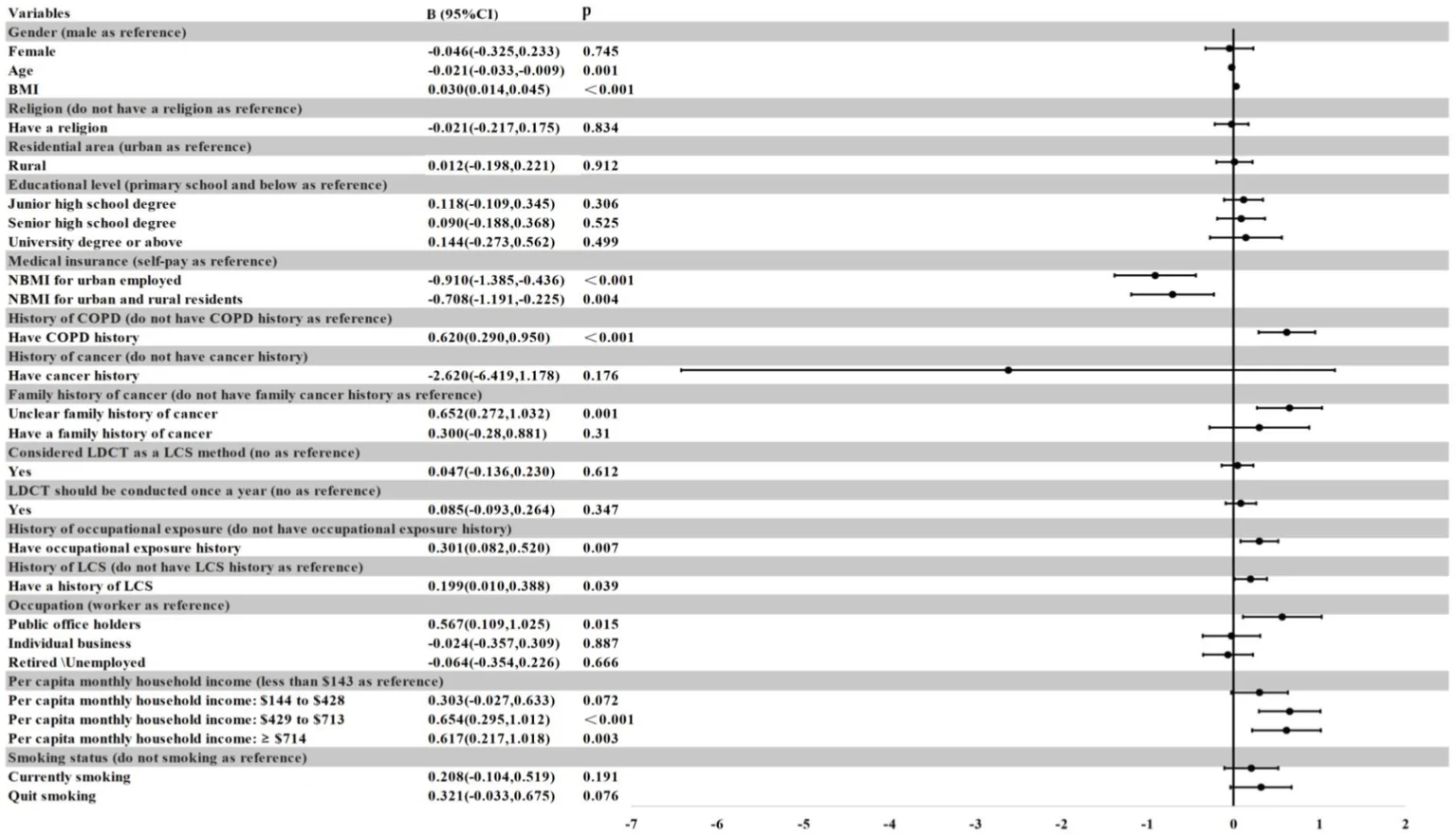
Forest plot showing the association between perceived risk of lung cancer and associated factors. *R^2^* = 0.119, adjusted *R^2^* = 0.107, *F* = 10.007, *p* < 0.001, the B reported is unstandardized regression coefficients.

**Figure 6:**
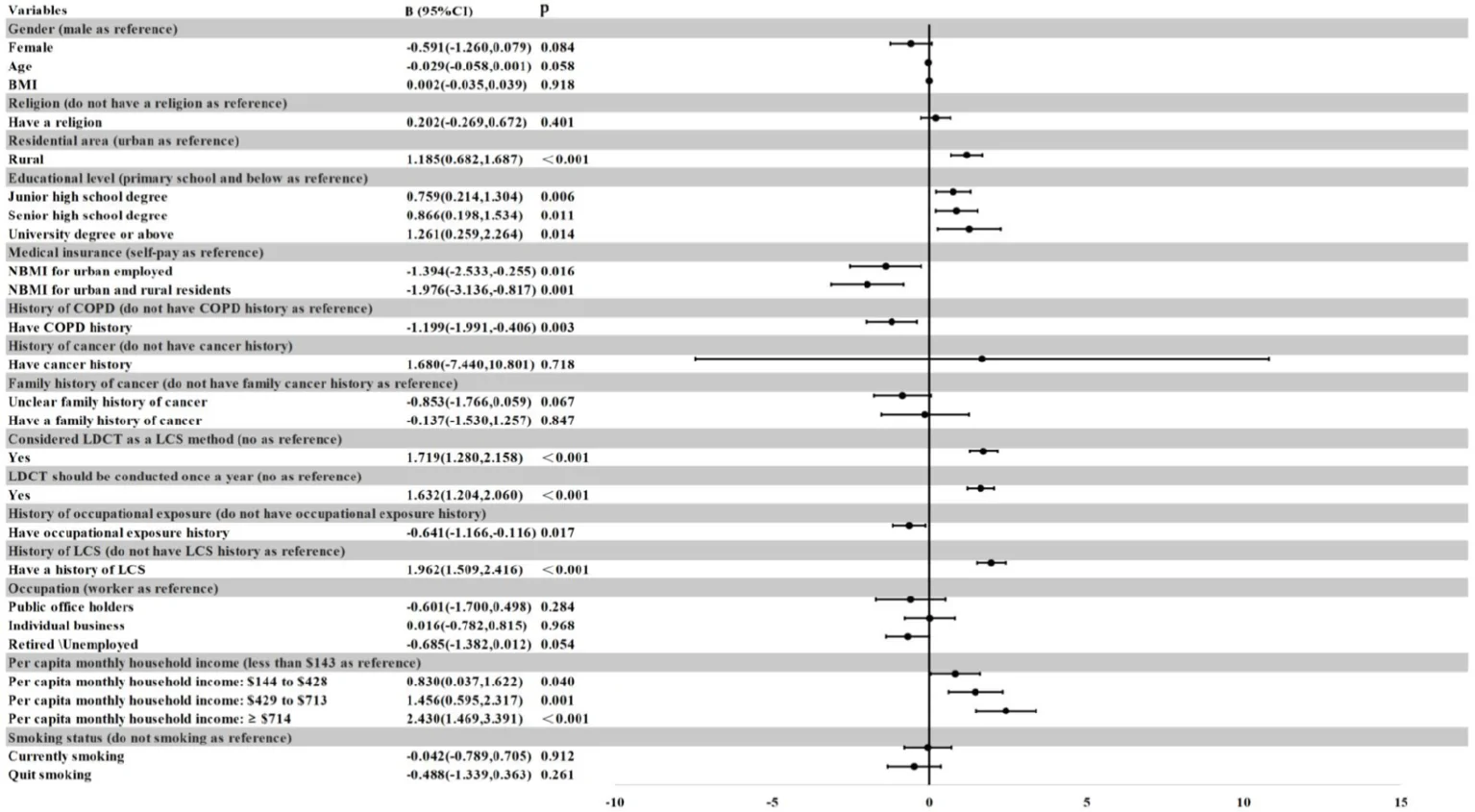
Forest plot showing the association between self-efficacy of LCS and associated factors. *R^2^* = 0.178, adjusted *R^2^* = 0.167, *F* = 16.048, *p* < 0.001, the B reported is unstandardized regression coefficients.

## 4 Discussion

This study examined LCS health beliefs and their associated factors among individuals at high risk for lung cancer in Fujian Province, China. Several sociodemographic, health-related, and LCS-related characteristics were associated with overall and domain-specific LCS health belief. In particular, LCS-related knowledge and previous screening experience were associated with more favorable health belief scores, whereas associations with sociodemographic and health-related characteristics varied across different dimensions of health beliefs. These findings extend the limited evidence on LCS health beliefs among high-risk populations in China and may inform future strategies to support informed participation in LCS.

Participants who correctly identified LDCT as the recommended LCS method and recognized the recommended screening frequency, as well as those with a history of LCS, had more favorable LCS health belief scores. This finding is broadly consistent with the knowledge-attitude-practice framework, which suggests close relationships among health knowledge, attitudes, and practices (37). Greater knowledge of LCS may be associated with a clearer understanding of its purpose, potential benefits, and recommended procedures, although the cross-sectional design does not allow the direction of this relationship to be established. Similarly, previous screening experience may provide greater familiarity with the screening process and opportunities for communication with healthcare professionals, which may be related to more favorable screening beliefs. However, previous studies have suggested that negative screening results may sometimes create “false reassurance,” thereby reducing perceived susceptibility to lung cancer and motivation for subsequent screening (38). Thus, previous screening experience may be associated with health beliefs in different ways depending on how individuals understand their screening results and personal risk. These findings highlight the potential value of clear risk communication and appropriate explanation of screening results throughout the LCS process to support informed decisions regarding subsequent screening.

Consistent with previous studies, higher BMI, religious affiliation, higher educational level, and higher household income were associated with more favorable scores on some dimensions of LCS health beliefs (39, 40). However, the mechanisms underlying these associations cannot be determined from the present study. For example, the association with BMI may reflect differences in health awareness, perceived health needs, or healthcare contact (41), whereas the association with religious affiliation may potentially relate to social support and social networks associated with religious participation (42–44). Because health awareness, personality characteristics, and religion-related social support were not directly measured, these explanations remain hypothetical and should not be interpreted as mechanisms demonstrated by the present findings. Higher educational level and income may similarly be associated with greater access to health information, healthcare resources, and opportunities to engage with screening services, which may partly explain their associations with perceived benefits and self-efficacy regarding LCS (45, 46). Future studies incorporating direct measures of health literacy, healthcare access, and social support are needed to clarify these relationships.

Female participants and current or former smokers had less favorable scores on some dimensions of LCS health beliefs. Previous studies have similarly shown that objective lung cancer risk does not necessarily correspond to perceived susceptibility, even among individuals who meet high-risk criteria of lung cancer (47, 48). Among smokers, smoking-related stigma has been associated with perceived barriers to LCS and lower engagement with healthcare services (26, 49–54). Although smoking-related stigma was not measured in the present study and therefore cannot be established as an explanation for the observed association, it may be an important psychosocial factor to examine in future research. Screening communication for people with a smoking history may therefore benefit from nonjudgmental and supportive approaches that facilitate informed consideration of LCS.

The observed associations with sex should also be interpreted cautiously. Differences in smoking patterns, awareness of non-smoking-related lung cancer risk factors, perceived susceptibility, caregiving responsibilities, and psychological responses to screening have been reported between women and men (54–57). However, these factors were not directly assessed as mechanisms in the present study, and the lower health belief scores observed among women cannot be attributed to any single explanation. In particular, lower smoking prevalence among women does not necessarily indicate low lung cancer risk, because other risk factors may also be relevant. Future research should directly examine sex-related differences in risk perception, knowledge of lung cancer risk factors, practical barriers, and screening-related concerns. From a practice perspective, LCS education may need to address both smoking- and non-smoking-related risk factors rather than relying predominantly on smoking-focused risk messages.

Participation in the national basic medical insurance program was associated with less favorable LCS health belief scores in some models. Although basic medical insurance provides broad healthcare coverage in China, insurance status may also be related to socioeconomic circumstances, healthcare accessibility, and the affordability of screening services (57, 58). Because the present study did not directly assess LCS-specific insurance coverage, out-of-pocket costs, or access to LDCT services, the observed association should not be interpreted as an effect of insurance itself. Rather, it suggests the need for future research to examine how insurance coverage, affordability, socioeconomic circumstances, and healthcare access jointly relate to LCS beliefs and screening decisions.

Associations involving age, COPD history, and occupational exposure were less consistent across the overall and domain-specific LCS health belief scores, which is broadly consistent with the heterogeneous findings reported in previous studies. Although older age and conditions or exposures associated with elevated lung cancer risk might be expected to correspond to greater perceived susceptibility, objective risk does not necessarily translate directly into subjective risk perception. Differences in risk awareness, healthcare experience, and access to health information may contribute to these patterns, but these potential explanations were not directly assessed in the present study. Accordingly, culturally specific mechanisms such as filial support should not be inferred from the present findings without direct measurement. Future studies should examine how age-related risk knowledge, family support, and healthcare experiences are related to LCS beliefs among older high-risk adults.

Similarly, COPD and occupational exposure are recognized indicators of increased lung cancer risk, yet their associations with screening-related beliefs may depend on whether individuals recognize these conditions or exposures as relevant to their personal lung cancer risk. Previous research has suggested that prolonged exposure to health risks may, in some contexts, be accompanied by normalization of risk (48, 59). However, risk normalization was not measured in this study and therefore cannot be inferred as an explanation for the observed associations. Future studies should directly assess perceived susceptibility, knowledge of exposure-related risk, and risk normalization to clarify how objective risk characteristics relate to LCS health beliefs.

Finally, associations involving residential area and occupation varied across the overall and domain-specific models. Rural–urban differences in LCS health beliefs may reflect multiple contextual factors, including differences in socioeconomic conditions, healthcare accessibility, health information exposure, and local screening resources. Similarly, broad occupational categories may encompass heterogeneous working conditions and health-related exposures. Because these contextual factors were not comprehensively measured, the observed associations cannot be attributed to specific mechanisms. Further studies incorporating more detailed measures of residential context, occupational characteristics, and healthcare accessibility are needed to clarify these relationships.

There are several limitations to this study. First, the cross-sectional design precludes establishing temporal precedence or causal relationships, and reverse causation cannot be excluded. Accordingly, the observed relationships should be interpreted as associations rather than evidence that the identified characteristics influence or determine LCS health beliefs.

Second, although a four-stage multistage sampling procedure incorporating PPS sampling was used, the regression analyses did not account for sampling weights, design effects, or within-cluster correlations. Participants recruited from the same community or village may share characteristics, potentially violating the independence assumption of conventional regression models. Failure to account for clustering may have resulted in underestimated standard errors and overly precise confidence intervals, while the absence of sampling weights may limit the population representativeness of the estimates. The regression findings should therefore be interpreted as individual level associations rather than design-weighted population estimates. Future studies should retain detailed sampling information and use survey-weighted or multilevel analytical approaches to appropriately account for complex sampling designs.

Third, nonresponse may have introduced selection bias if individuals who did not complete the survey differed systematically from those included in the analysis. In addition, electronic questionnaire administration may have preferentially included individuals with greater access to or familiarity with digital technology, although trained researchers provided assistance when needed.

Fourth, participants were recruited from only Fuzhou and Putian in Fujian Province. Although both urban and rural populations were included, geographic, socioeconomic, healthcare-system, and cultural differences may limit the generalizability of the findings to other regions of China.

Fifth, the study relied primarily on self-reported measures, which may be subject to recall and social desirability biases. Future studies could incorporate objective clinical and screening data where appropriate.

Sixth, multiple statistical tests were conducted without formal adjustment for multiple comparisons, which may have increased the risk of Type I error. The findings, particularly associations observed only for individual subscales, should therefore be interpreted cautiously. Future studies may consider prespecifying primary hypotheses or applying appropriate procedures to address multiple testing.

Seventh, although multiple sociodemographic and health-related covariates were included, residual confounding and unmeasured factors cannot be excluded. Potentially relevant constructs, including cancer fatalism, medical trust, media exposure, social influence, lung cancer fear and worry, health literacy, and smoking-related stigma, were not directly assessed (29). Their proposed roles in explaining the observed associations therefore remain hypothetical and should be examined directly in future studies.

Eighth, the eligibility criteria were based on the LCS guideline applied at the time of the study. Risk profiles not fully captured by conventional age- and smoking-based criteria, including some risks relevant to never-smokers, may therefore have been underrepresented. Future research could examine whether LCS health beliefs differ when broader risk-prediction approaches or updated screening eligibility criteria are applied.

Finally, data were collected between 2020 and 2022, during the COVID-19 pandemic, when healthcare access, resource availability, and screening-related perceptions may have differed from those in the post-pandemic period. The temporal context should therefore be considered when interpreting the findings, and further research is needed to examine their applicability in current healthcare settings.

## 5 Conclusion

This study identified several sociodemographic, health-related, and LCS-related characteristics associated with LCS health beliefs among individuals at high risk for lung cancer. In particular, LCS-related knowledge and previous screening experience were associated with more favorable health belief scores, while associations with sociodemographic and health-related characteristics varied across different dimensions of health beliefs. These findings highlight the importance of considering differences in LCS knowledge, screening experience, and socioeconomic and health-related characteristics when developing future LCS education and support strategies. Such strategies may help address screening-related beliefs and support informed participation in LCS among high-risk populations.

## Data Availability

The raw data supporting the conclusions of this article will be made available by the authors, without undue reservation.

## Footnotes

1
https://www.wjx.cn/vm/QmuaKRb.aspx
