# Supplementary material for: Lung cancer screening health beliefs among a high-risk population in China: a cross-sectional survey
Source: Front Public Health. 2026 Oct 2;14:1759430. doi: 10.3389/fpubh.2026.1759430 (PMC13672327; doi:10.3389/fpubh.2026.1759430)
Supplement: Supplementary file 1 [file Data_Sheet_1.docx]

**Appendix 1** Table 1. Multiple Linear Regression Analyses of Factors Associated With LCS Health Beliefs

| **Variable** | **B** | **SE** | **p-value** | **95 % CI** |
| --- | --- | --- | --- | --- |
| **Male** | **Ref** |  |  |  |
| Female | -1.627 | 0.627 | 0.010 | -2.856, -0.398 |
| **Age** | 0.057 | 0.057 | 0.039 | 0.003, 0.112 |
| **BMI (kg/㎡)** | 0.018 | 0.018 | 0.600 | -0.050, 0.087 |
| **Religion: No** | **Ref** |  |  |  |
| Religion: Yes | 0.917 | 0.917 | 0.037 | 0.053, 1.781 |
| **Residential area: Urban** | **Ref** |  |  |  |
| Residential area: Rural | 3.276 | 0.470 | ＜0.001 | 2.353, 4.199 |
| **History of** **COPD: No** | **Ref** |  |  |  |
| History of COPD: Yes | -4.820 | 0.742 | ＜0.001 | -6.275, -3.365 |
| **Considered** **LDCT as a LCS method: No** | **Ref** |  |  |  |
| Considered LDCT as a LCS method: Yes | 4.912 | 0.411 | ＜0.001 | 4.106, 5.719 |
| **LDCT should be conducted once a year: No** | **Ref** |  |  |  |
| LDCT should be conducted once a year: Yes | 4.640 | 0.401 | ＜0.001 | 3.854, 5.425 |
| **History of occupational exposure: No** | **Ref** |  |  |  |
| History of occupational exposure: Yes | -0.911 | 0.491 | 0.064 | -1.874, 0.053 |
| **History of LCS: No** | **Ref** |  |  |  |
| History of LCS: Yes | 5.717 | 0.424 | ＜0.001 | 4.885, 6.548 |
| **Educational level: Primary school and below** | **Ref** |  |  |  |
| Educational level: junior high school degree | 0.698 | 0.510 | 0.171 | -0.302, 1.698 |
| Educational level: senior high school degree | 1.120 | 0.625 | 0.073 | -0.106, 2.345 |
| Educational level: university degree or above | 1.187 | 0.938 | 0.206 | -0.652, 3.027 |
| **Medical insurance: Self-pay** | **Ref** |  |  |  |
| Medical insurance: NBMI for urban employed | -2.894 | 1.066 | 0.007 | -4.984, -0.805 |
| Medical insurance: NBMI for urban and rural residents | -4.122 | 1.085 | ＜0.001 | -6.250, -1.994 |
| **Family history of cancer: No** | **Ref** |  |  |  |
| Family history of cancer: unclear | -1.212 | 0.854 | 0.156 | -2.886, 0.462 |
| Family history of cancer: yes | 0.632 | 1.304 | 0.628 | -1.925, 3.189 |
| **Occupation: Worker** | **Ref** |  |  |  |
| Occupation: Civil servant | -1.640 | 1.028 | 0.111 | -3.657, 0.376 |
| Occupation: Businessperson | -0.914 | 0.747 | 0.222 | -2.379, 0.552 |
| Occupation:Retired /Unemployed | -0.309 | 0.652 | 0.636 | -1.589, 0.970 |
| **Per capita monthly household income: Less than $143** | **Ref** |  |  |  |
| Per capita monthly household income: $144 to $428 | 1.917 | 0.741 | 0.010 | 0.463, 3.371 |
| Per capita monthly household income: $429 to $713 | 3.142 | 0.806 | ＜0.001 | 1.562, 4.722 |
| Per capita monthly household income: ≥ $714 | 3.586 | 0.899 | ＜0.001 | 1.823, 5.349 |
| **Smoking status: No** | **Ref** |  |  |  |
| Smoking status: Currently smoking | -1.722 | 0.699 | 0.014 | -3.093, -0.351 |
| Smoking status: Quit smoking | -1.802 | 0.796 | 0.024 | -3.363, -0.241 |
| ***R^2^*** | 0.312 |  |  |  |
| **Adjusted *R^2^*** | 0.303 |  |  |  |
| ***F*** | 33.648 |  |  |  |
| **Durbin Watson** | 0.562 |  |  |  |

**Appendix 1** Table 2. Multiple Linear Regression Analyses of Factors Associated With Perceived Barriers of Lung Cancer Screening

| **Variable** | **B** | **SE** | **p-value** | **95 % CI** |
| --- | --- | --- | --- | --- |
| **Male** | **Ref** |  |  |  |
| Female | 0.903 | 0.430 | 0.036 | -1.747, -0.059 |
| **Age** | 0.127 | 0.019 | ＜0.001 | 0.089, 0.164 |
| **BMI (kg/㎡)** | -0.012 | 0.024 | 0.620 | -0.059, 0.035 |
| **Religion: No** | **Ref** |  |  |  |
| Religion: Yes | 0.882 | 0.302 | 0.004 | 0.288, 1.475 |
| **Residential area: Urban** | **Ref** |  |  |  |
| Residential area: Rural | 1.996 | 0.323 | ＜0.001 | 1.363, 2.630 |
| **History of COPD: No** | **Ref** |  |  |  |
| History of COPD: Yes | -4.187 | 0.509 | ＜0.001 | -5.186, -3.188 |
| **Considered LDCT as a LCS method: No** | **Ref** |  |  |  |
| Considered LDCT as a LCS method: Yes | 2.215 | 0.282 | ＜0.001 | 1.662, 2.769 |
| **LDCT should be conducted once a year: No** | **Ref** |  |  |  |
| LDCT should be conducted once a year: Yes | 2.313 | 0.275 | ＜0.001 | 1.773, 2.852 |
| **History of occupational exposure: No** | **Ref** |  |  |  |
| History of occupational exposure: Yes | -0.268 | 0.337 | 0.427 | -0.930, 0.394 |
| **History of LCS: No** | **Ref** |  |  |  |
| History of LCS: Yes | 2.727 | 0.291 | ＜0.001 | 2.155, 3.298 |
| **Educational level: Primary school and below** | **Ref** |  |  |  |
| Educational level: junior high school degree | -0.520 | 0.350 | 0.138 | -1.207, 0.167 |
| Educational level: senior high school degree | -0.350 | 0.429 | 0.415 | -1.192, 0.492 |
| Educational level: university degree or above | -1.098 | 0.644 | 0.088 | -2.362, 0.165 |
| **Medical insurance: Self-pay** | **Ref** |  |  |  |
| Medical insurance: NBMI for urban employed | -0.572 | 0.732 | 0.434 | -2.007, 0.863 |
| Medical insurance: NBMI for urban and rural residents | -1.674 | 0.745 | 0.025 | -3.135, -0.212 |
| **Family history of cancer: No** | **Ref** |  |  |  |
| Family history of cancer: unclear | -0.939 | 0.586 | 0.109 | -2.089, 0.211 |
| Family history of cancer: yes | 0.751 | 0.895 | 0.402 | -1.005, 2.508 |
| **Occupation: Worker** | **Ref** |  |  |  |
| Occupation: Civil servant | -0.786 | 0.706 | 0.265 | -2.171, 0.599 |
| Occupation: Businessperson | -0.630 | 0.513 | 0.220 | -1.636, 0.377 |
| Occupation:Retired \Unemployed | 0.241 | 0.448 | 0.591 | -0.638, 1.120 |
| **Per capita monthly household income: Less than $143** | **Ref** |  |  |  |
| Per capita monthly household income: $144 to $428 | 0.479 | 0.590 | 0.347 | -0.520, 1.477 |
| Per capita monthly household income: $429 to $713 | 0.910 | 0.553 | 0.100 | -0.175, 1.995 |
| Per capita monthly household income: ≥ $714 | 0.455 | 0.617 | 0.461 | -0.756, 1.666 |
| **Smoking status: No** | **Ref** |  |  |  |
| Smoking status: Currently smoking | -1.925 | 0.480 | ＜0.001 | -2.866, -0.983 |
| Smoking status: Quit smoking | -1.516 | 0.547 | ＜0.001 | -2.588, -0.444 |
| ***R^2^*** | 0.272 |  |  |  |
| **Adjusted *R^2^*** | 0.262 |  |  |  |
| ***F*** | 27.694 |  |  |  |
| **Durbin Watson** | 1.098 |  |  |  |

**Appendix 1	Table 3.** Multiple Linear Regression Analyses of Factors Associated With Perceived Benefit of Lung Cancer Screening

| **Variable** | **B** | **SE** | **p-value** | **95 % CI** |
| --- | --- | --- | --- | --- |
| **Male** | **Ref** |  |  |  |
| Female | -0.087 | 0.193 | 0.652 | -0.466, 0.292 |
| **Age** | -0.020 | 0.009 | 0.022 | -0.036, -0.003 |
| **BMI (kg/㎡)** | -0.002 | 0.011 | 0.875 | -0.023, 0.019 |
| **Religion: No** | **Ref** |  |  |  |
| Religion: Yes | -0.145 | 0.136 | 0.285 | -0.412, 0.121 |
| **Residential area: Urban** | **Ref** |  |  |  |
| Residential area: Rural | 0.083 | 0.145 | 0.565 | -0.201,0.368 |
| **History of COPD: No** | **Ref** |  |  |  |
| History of COPD: Yes | -0.055 | 0.229 | 0.811 | -0.503, 0.394 |
| **Considered LDCT as a LCS method: No** | **Ref** |  |  |  |
| Considered LDCT as a LCS method: Yes | 0.931 | 0.127 | ＜0.001 | 0.682, 1.179 |
| **LDCT should be conducted once a year: No** | **Ref** |  |  |  |
| LDCT should be conducted once a year: Yes | 0.610 | 0.123 | ＜0.001 | 0.368, 0.852 |
| **History of occupational exposure: No** | **Ref** |  |  |  |
| History of occupational exposure: Yes | -0.302 | 0.151 | 0.046 | -0.599, -0.005 |
| **History of LCS: No** | **Ref** |  |  |  |
| History of LCS: Yes | 0.829 | 0.131 | ＜0.001 | 0.572, 1.085 |
| **Educational level: Primary school and below** | **Ref** |  |  |  |
| Educational level: junior high school degree | 0.341 | 0.157 | 0.030 | 0.032, 0.649 |
| Educational level: senior high school degree | 0.513 | 0.193 | 0.008 | 0.135, 0.891 |
| Educational level: university degree or above | 0.880 | 0.289 | 0.002 | 0.313, 1.448 |
| **Medical insurance: Self-pay** | **Ref** |  |  |  |
| Medical insurance: NBMI for urban employed | -0.018 | 0.328 | 0.957 | -0.662, 0.626 |
| Medical insurance: NBMI for urban and rural residents | 0.235 | 0.335 | 0.482 | -0.421, 0.892 |
| **Family history of cancer: No** | **Ref** |  |  |  |
| Family history of cancer: unclear | -0.071 | 0.263 | 0.786 | -0.588, 0.445 |
| Family history of cancer: yes | -0.283 | 0.402 | 0.482 | -1.071, 0.505 |
| **Occupation: Worker** | **Ref** |  |  |  |
| Occupation: Civil servant | -0.821 | 0.317 | 0.010 | -1.442, -0.199 |
| Occupation: Businessperson | -0.276 | 0.230 | 0.231 | -0.728, 0.176 |
| Occupation:Retired \Unemployed | 0.199 | 0.201 | 0.324 | -0.196, 0.593 |
| **Per capita monthly household income: Less than $143** | **Ref** |  |  |  |
| Per capita monthly household income: $144 to $428 | 0.305 | 0.229 | 0.182 | -0.143, 0.753 |
| Per capita monthly household income: $429 to $713 | 0.122 | 0.248 | 0.624 | -0.365, 0.609 |
| Per capita monthly household income: ≥ $714 | 0.083 | 0.277 | 0.764 | -0.460, 0.627 |
| **Smoking status: No** | **Ref** |  |  |  |
| Smoking status: Currently smoking | 0.037 | 0.215 | 0.864 | -0.386, 0.460 |
| Smoking status: Quit smoking | -0.119 | 0.245 | 0.627 | -0.600, 0.362 |
| ***R^2^*** | 0.096 |  |  |  |
| **Adjusted *R^2^*** | 0.084 |  |  |  |
| ***F*** | 7.876 |  |  |  |
| **Durbin Watson** | 1.453 |  |  |  |

**Appendix 1	Table 4.** Multiple Linear Regression Analyses of Factors Associated With Perceived Risk of Lung Cancer

| **Variable** | **B** | **SE** | **p-value** | **95 % CI** |
| --- | --- | --- | --- | --- |
| **Male** | **Ref** |  |  |  |
| Female | -0.046 | 0.142 | 0.745 | -0.325, 0.233 |
| **Age** | -0.021 | 0.006 | 0.001 | -0.033, -0.009 |
| **BMI (kg/㎡)** | 0.030 | 0.008 | ＜0.001 | 0.014, 0.045 |
| **Religion: No** | **Ref** |  |  |  |
| Religion: Yes | -0.021 | 0.100 | 0.834 | -0.217, 0.175 |
| **Residential area: Urban** | **Ref** |  |  |  |
| Residential area: Rural | 0.012 | 0.107 | 0.912 | -0.198, 0.221 |
| **History of COPD: No** | **Ref** |  |  |  |
| History of COPD: Yes | 0.620 | 0.168 | 0.290 | 0.290, 0.950 |
| **Considered LDCT as a LCS method: No** | **Ref** |  |  |  |
| Considered LDCT as a LCS method: Yes | 0.047 | 0.093 | 0.612 | -0.136, 0.230 |
| **LDCT should be conducted once a year: No** | **Ref** |  |  |  |
| LDCT should be conducted once a year: Yes | 0.085 | 0.091 | 0.347 | -0.093, 0.264 |
| **History of occupational exposure: No** | **Ref** |  |  |  |
| History of occupational exposure: Yes | 0.301 | 0.112 | 0.007 | 0.082, 0.520 |
| **History of LCS: No** | **Ref** |  |  |  |
| History of LCS: Yes | 0.199 | 0.096 | 0.039 | 0.010, 0.388 |
| **Educational level: Primary school and below** | **Ref** |  |  |  |
| Educational level: junior high school degree | 0.118 | 0.116 | 0.306 | -0.109, 0.345 |
| Educational level: senior high school degree | 0.090 | 0.142 | 0.525 | -0.188, 0.368 |
| Educational level: university degree or above | 0.144 | 0.213 | 0.499 | -0.273, 0.562 |
| **Medical insurance: Self-pay** | **Ref** |  |  |  |
| Medical insurance: NBMI for urban employed | -0.910 | 0.242 | ＜0.001 | -1.385, -0.436 |
| Medical insurance: NBMI for urban and rural residents | -0.708 | 0.246 | 0.004 | -1.191, -0.225 |
| **Family history of cancer: No** | **Ref** |  |  |  |
| Family history of cancer: unclear | 0.652 | 0.194 | 0.001 | 0.272, 1.032 |
| Family history of cancer: yes | 0.300 | 0.296 | 0.310 | -0.280, 0.881 |
| **Occupation: Worker** | **Ref** |  |  |  |
| Occupation: Civil servant | 0.567 | 0.233 | 0.015 | 0.109, 1.025 |
| Occupation: Businessperson | -0.024 | 0.170 | 0.887 | -0.357, 0.309 |
| Occupation:Retired \Unemployed | -0.064 | 0.148 | 0.666 | -0.354, 0.226 |
| **Per capita monthly household income: Less than $143** | **Ref** |  |  |  |
| Per capita monthly household income: $144 to $428 | 0.303 | 0.168 | 0.072 | -0.027, 0.633 |
| Per capita monthly household income: $429 to $713 | 0.654 | 0.183 | ＜0.001 | 0.295, 1.012 |
| Per capita monthly household income: ≥ $714 | 0.617 | 0.204 | 0.003 | 0.217, 1.018 |
| **Smoking status: No** | **Ref** |  |  |  |
| Smoking status: Currently smoking | 0.208 | 0.159 | 0.191 | -0.104, 0.519 |
| Smoking status: Quit smoking | 0.321 | 0.181 | 0.076 | -0.033, 0.675 |
| ***R^2^*** | 0.119 |  |  |  |
| **Adjusted *R^2^*** | 0.107 |  |  |  |
| ***F*** | 10.007 |  |  |  |
| **Durbin Watson** | 1.640 |  |  |  |

**Appendix 1	Table 5.** Multiple Linear Regression Analyses of Factors Associated With Perceived Self-efficacy of Lung Cancer Screening

| **Variable** | **B** | **SE** | **p-value** | **95 % CI** |
| --- | --- | --- | --- | --- |
| **Male** | **Ref** |  |  |  |
| Female | -0.591 | 0.342 | 0.084 | -1.260, 0.079 |
| **Age** | -0.029 | 0.015 | 0.058 | -0.058, 0.001 |
| **BMI (kg/㎡)** | 0.002 | 0.019 | 0.918 | -0.035, 0.039 |
| **Religion: No** | **Ref** |  |  |  |
| Religion: Yes | 0.202 | 0.240 | 0.401 | -0.269, 0.672 |
| **Residential area: Urban** | **Ref** |  |  |  |
| Residential area: Rural | 1.185 | 0.256 | ＜0.001 | 0.682, 1.687 |
| **History of COPD: No** | **Ref** |  |  |  |
| History of COPD: Yes | -1.199 | 0.404 | 0.003 | -1.991, -0.406 |
| **Considered LDCT as a LCS method: No** | **Ref** |  |  |  |
| Considered LDCT as a LCS method: Yes | 1.719 | 0.224 | ＜0.001 | 1.280, 2.158 |
| **LDCT should be conducted once a year: No** | **Ref** |  |  |  |
| LDCT should be conducted once a year: Yes | 1.632 | 0.218 | ＜0.001 | 1.204, 2.060 |
| **History of occupational exposure: No** | **Ref** |  |  |  |
| History of occupational exposure: Yes | -0.641 | 0.268 | 0.017 | -1.166, -0.116 |
| **History of LCS: No** | **Ref** |  |  |  |
| History of LCS: Yes | 1.962 | 0.231 | ＜0.001 | 1.509, 2.416 |
| **Educational level: Primary school and below** | **Ref** |  |  |  |
| Educational level: junior high school degree | 0.759 | 0.278 | 0.006 | 0.214, 1.304 |
| Educational level: senior high school degree | 0.866 | 0.341 | 0.011 | 0.198, 1.534 |
| Educational level: university degree or above | 1.261 | 0.511 | 0.014 | 0.259, 2.264 |
| **Medical insurance: Self-pay** | **Ref** |  |  |  |
| Medical insurance: NBMI for urban employed | -1.394 | 0.581 | 0.016 | -2.533, -0.255 |
| Medical insurance: NBMI for urban and rural residents | -1.976 | 0.591 | 0.001 | -3.136, -0.817 |
| **Family history of cancer: No** | **Ref** |  |  |  |
| Family history of cancer: unclear | -0.853 | 0.465 | 0.067 | -1.766, 0.059 |
| Family history of cancer: yes | -0.137 | 0.711 | 0.847 | -1.530, 1.257 |
| **Occupation: Worker** | **Ref** |  |  |  |
| Occupation: Civil servant | -0.601 | 0.560 | 0.284 | -1.700, 0.498 |
| Occupation: Businessperson | 0.016 | 0.407 | 0.968 | -0.782, 0.815 |
| Occupation:Retired \Unemployed | -0.685 | 0.356 | 0.054 | -1.382, 0.012 |
| **Per capita monthly household income: Less than $143** | **Ref** |  |  |  |
| Per capita monthly household income: $144 to $428 | 0.830 | 0.404 | 0.040 | 0.037, 1.622 |
| Per capita monthly household income: $429 to $713 | 1.456 | 0.439 | 0.001 | 0.595, 2.317 |
| Per capita monthly household income: ≥ $714 | 2.430 | 0.490 | ＜0.001 | 1.469, 3.391 |
| **Smoking status: No** | **Ref** |  |  |  |
| Smoking status: Currently smoking | -0.042 | 0.381 | 0.912 | -0.789, 0.705 |
| Smoking status: Quit smoking | -0.488 | 0.434 | 0.261 | -1.339, 0.363 |
| ***R^2^*** | 0.178 |  |  |  |
| **Adjusted *R^2^*** | 0.167 |  |  |  |
| ***F*** | 16.048 |  |  |  |
| **Durbin Watson** | 1.050 |  |  |  |

**Appendix 2** Table 1. Variance Inflation Factor and Tolerance Values for Independent Variables

| **Variables** | **Tolerance** | **VIF** |
| --- | --- | --- |
| **Gender: Male** |  | |
| Female | **0.386** | **2.592** |
| **Age** | 0.596 | 1.679 |
| **BMI (kg/㎡)** | 0.913 | 1.095 |
| **Religion: No** |  | |
| Religion: Yes | 0.826 | 1.211 |
| **Residential area: Urban** |  | |
| Residential area: Rural | 0.664 | 1.506 |
| **History of COPD: No** |  | |
| History of COPD: Yes | 0.906 | 1.104 |
| **History of lung cancer (do not have a cancer history)** |  | |
| **Have cancer history** | 0.983 | 1.017 |
| **Considered LDCT as a LCS method: No** |  | |
| Considered LDCT as a LCS method: Yes | 0.926 | 1.080 |
| **LDCT should be conducted once a year: No** |  | |
| LDCT should be conducted once a year: Yes | **0.917** | **1.091** |
| **History of occupational exposure: No** |  | |
| History of occupational exposure: Yes | 0.826 | 1.210 |
| **History of LCS: No** |  | |
| History of LCS: Yes | 0.829 | 1.206 |
| **Educational level: Primary school and below** |  | |
| Educational level: junior high school degree | 0.660 | 1.516 |
| Educational level: senior high school degree | 0.495 | 2.021 |
| Educational level: university degree or above | 0.591 | 1.693 |
| **Medical insurance: Self-pay** |  | |
| Medical insurance: NBMI for urban employed | 0.139 | 7.202 |
| Medical insurance: NBMI for urban and rural residents | 0.129 | 7.753 |
| **Family history of cancer: No** |  | |
| Family history of cancer: unclear | 0.900 | 1.112 |
| Family history of cancer: yes | 0.978 | 1.022 |
| **Occupation: Worker** |  | |
| Occupation: Civil servant | 0.615 | 1.626 |
| Occupation: Businessperson | 0.698 | 1.433 |
| Occupation:Retired \Unemployed | 0.368 | 2.719 |
| **Per capita monthly household income: Less than $143** |  | |
| Per capita monthly household income: $144 to $428 | 0.277 | 3.612 |
| Per capita monthly household income: $429 to $713 | 0.250 | 4.005 |
| Per capita monthly household income: ≥ $714 | 0.320 | 3.129 |
| **Smoking status: No** |  | |
| Smoking status: Currently smoking | 0.307 | 3.262 |
| Smoking status: Quit smoking | 0.508 | 1.970 |

**Appendix 2** Figure 1. Scatterplot of The Chinese version of the Lung Cancer Screening Health Belief Scales

**Appendix 2** Figure 2. Histogram of The Chinese version of the Lung Cancer Screening Health Belief Scales

## Appendix 3 Table 1. Multiple Linear Regression Analysis of Factors Associated with Perceived Barriers to Lung Cancer Screening (HC3 analysis)

| **Variable** | **B** | **Robust SE** | **t** | **p** | **95% CI** |
| --- | --- | --- | --- | --- | --- |
| Intercept | 35.676 | 2.130 | 16.746 | <0.001 | 31.498, 39.854 |
| **Sex** (ref: Male) |  |  |  |  |  |
| Female | -0.903 | 0.468 | -1.929 | 0.054 | -1.821, 0.015 |
| Age | 0.127 | 0.020 | 6.345 | <0.001 | 0.088, 0.166 |
| BMI | -0.012 | 0.031 | -0.378 | 0.706 | -0.073, 0.050 |
| **Religion** (ref: No) |  |  |  |  |  |
| Yes | 0.882 | 0.301 | 2.928 | 0.003 | 0.291, 1.472 |
| **Residential area** (ref: Urban) |  |  |  |  |  |
| Rural | 1.996 | 0.343 | 5.824 | <0.001 | 1.324, 2.668 |
| **Education level** (ref: Primary school and below) |  |  |  |  |  |
| Junior high school | -0.520 | 0.350 | -1.483 | 0.138 | -1.207, 0.167 |
| Senior high school | -0.350 | 0.425 | -0.823 | 0.410 | -1.183, 0.484 |
| University or above | -1.098 | 0.737 | -1.490 | 0.136 | -2.543, 0.347 |
| **Medical insurance** (ref: Self-pay) |  |  |  |  |  |
| NBMI for urban employed | -0.572 | 1.137 | -0.503 | 0.615 | -2.801, 1.658 |
| NBMI for urban and rural residents | -1.674 | 1.197 | -1.398 | 0.162 | -4.022, 0.675 |
| **History of COPD** (ref: No) |  |  |  |  |  |
| Yes | -4.187 | 0.640 | -6.539 | <0.001 | -5.443, -2.931 |
| **History of cancer** (ref: No) |  |  |  |  |  |
| Yes | 6.280 | 32.011 | 0.196 | 0.844 | -56.499, 69.059 |
| **Family history of cancer** (ref: No) |  |  |  |  |  |
| Uncertain | -0.939 | 0.687 | -1.367 | 0.172 | -2.287, 0.408 |
| Yes | 0.751 | 0.901 | 0.834 | 0.404 | -1.015, 2.517 |
| **Consider LDCT as LCS method** (ref: No) |  |  |  |  |  |
| Yes | 2.215 | 0.285 | 7.763 | <0.001 | 1.656, 2.775 |
| **LDCT should be conducted once a year** (ref: No) |  |  |  |  |  |
| Yes | 2.313 | 0.268 | 8.633 | <0.001 | 1.787, 2.838 |
| **History of occupational exposure** (ref: No) |  |  |  |  |  |
| Yes | -0.268 | 0.339 | -0.790 | 0.429 | -0.933, 0.397 |
| **History of LCS** (ref: No) |  |  |  |  |  |
| Yes | 2.727 | 0.287 | 9.497 | <0.001 | 2.164, 3.290 |
| **Occupation** (ref: Worker) |  |  |  |  |  |
| Civil servant | -0.786 | 0.809 | -0.971 | 0.331 | -2.373, 0.801 |
| Businessperson | -0.630 | 0.558 | -1.128 | 0.259 | -1.725, 0.465 |
| Retired / Unemployed | 0.241 | 0.467 | 0.516 | 0.606 | -0.675, 1.157 |
| **Per capita monthly income** (ref: <$143) |  |  |  |  |  |
| $144–$428 | 0.479 | 0.555 | 0.864 | 0.388 | -0.609, 1.566 |
| $429–$713 | 0.910 | 0.629 | 1.446 | 0.148 | -0.324, 2.144 |
| ≥$714 | 0.455 | 0.686 | 0.664 | 0.507 | -0.889, 1.799 |
| **Smoking status** (ref: No) |  |  |  |  |  |
| Currently smoking | -1.925 | 0.538 | -3.577 | <0.001 | -2.980, -0.869 |
| Quit smoking | -1.516 | 0.581 | -2.607 | 0.009 | -2.656, -0.376 |

## Appendix 3 Table 2. Multiple Linear Regression Analysis of Factors Associated with Perceived Benefits of Lung Cancer Screening (HC3 analysis)

| **Variable** | **B** | **Robust SE** | **t** | **p** | **95% CI** |
| --- | --- | --- | --- | --- | --- |
| Intercept | 17.907 | 0.989 | 18.102 | <0.001 | 15.967, 19.846 |
| **Sex** (ref: Male) |  |  |  |  |  |
| Female | -0.087 | 0.207 | -0.420 | 0.674 | -0.494, 0.319 |
| Age | -0.020 | 0.009 | -2.098 | 0.036 | -0.038, -0.001 |
| BMI | -0.002 | 0.014 | -0.118 | 0.906 | -0.030, 0.026 |
| **Religion** (ref: No) |  |  |  |  |  |
| Yes | -0.145 | 0.150 | -0.968 | 0.333 | -0.440, 0.149 |
| **Residential area** (ref: Urban) |  |  |  |  |  |
| Rural | 0.083 | 0.137 | 0.608 | 0.543 | -0.185, 0.352 |
| **Education level** (ref: Primary school and below) |  |  |  |  |  |
| Junior high school | 0.341 | 0.150 | 2.267 | 0.024 | 0.046, 0.635 |
| Senior high school | 0.513 | 0.184 | 2.783 | 0.005 | 0.151, 0.875 |
| University or above | 0.880 | 0.348 | 2.533 | 0.011 | 0.199, 1.562 |
| **Medical insurance** (ref: Self-pay) |  |  |  |  |  |
| NBMI for urban employed | -0.018 | 0.503 | -0.035 | 0.972 | -1.005, 0.969 |
| NBMI for urban and rural residents | 0.235 | 0.524 | 0.449 | 0.653 | -0.792, 1.263 |
| **History of COPD** (ref: No) |  |  |  |  |  |
| Yes | -0.055 | 0.322 | -0.170 | 0.865 | -0.685, 0.576 |
| **History of cancer** (ref: No) |  |  |  |  |  |
| Yes | -0.251 | 16.004 | -0.016 | 0.987 | -31.638, 31.137 |
| **Family history of cancer** (ref: No) |  |  |  |  |  |
| Uncertain | -0.071 | 0.305 | -0.234 | 0.815 | -0.670, 0.527 |
| Yes | -0.283 | 0.465 | -0.608 | 0.543 | -1.195, 0.629 |
| **Consider LDCT as LCS method** (ref: No) |  |  |  |  |  |
| Yes | 0.931 | 0.125 | 7.422 | <0.001 | 0.685, 1.177 |
| **LDCT should be conducted once a year** (ref: No) |  |  |  |  |  |
| Yes | 0.610 | 0.120 | 5.094 | <0.001 | 0.375, 0.845 |
| **History of occupational exposure** (ref: No) |  |  |  |  |  |
| Yes | -0.302 | 0.154 | -1.963 | 0.050 | -0.604, 0.000 |
| **History of LCS** (ref: No) |  |  |  |  |  |
| Yes | 0.829 | 0.127 | 6.527 | <0.001 | 0.580, 1.078 |
| **Occupation** (ref: Worker) |  |  |  |  |  |
| Civil servant | -0.821 | 0.410 | -2.003 | 0.045 | -1.624, -0.017 |
| Businessperson | -0.276 | 0.249 | -1.108 | 0.268 | -0.765, 0.213 |
| Retired / Unemployed | 0.199 | 0.215 | 0.925 | 0.355 | -0.222, 0.619 |
| **Per capita monthly income** (ref: <$143) |  |  |  |  |  |
| $144–$428 | 0.305 | 0.261 | 1.170 | 0.242 | -0.206, 0.817 |
| $429–$713 | 0.122 | 0.284 | 0.428 | 0.669 | -0.436, 0.680 |
| ≥$714 | 0.083 | 0.323 | 0.258 | 0.796 | -0.551, 0.717 |
| **Smoking status** (ref: No) |  |  |  |  |  |
| Currently smoking | 0.037 | 0.224 | 0.165 | 0.869 | -0.402, 0.476 |
| Quit smoking | -0.119 | 0.252 | -0.473 | 0.636 | -0.613, 0.375 |

## Appendix 3 Table 3. Multiple Linear Regression Analysis of Factors Associated with Perceived Risk of Lung Cancer (HC3 analysis)

| **Variable** | **B** | **Robust SE** | **t** | **p** | **95% CI** |
| --- | --- | --- | --- | --- | --- |
| Intercept | 5.958 | 0.654 | 9.108 | <0.001 | 4.675, 7.241 |
| **Sex** (ref: Male) |  |  |  |  |  |
| Female | -0.046 | 0.139 | -0.334 | 0.738 | -0.319, 0.226 |
| Age | -0.021 | 0.007 | -3.203 | 0.001 | -0.034, -0.008 |
| BMI | 0.030 | 0.009 | 3.260 | 0.001 | 0.012, 0.048 |
| **Religion** (ref: No) |  |  |  |  |  |
| Yes | -0.021 | 0.098 | -0.213 | 0.832 | -0.214, 0.172 |
| **Residential area** (ref: Urban) |  |  |  |  |  |
| Rural | 0.012 | 0.108 | 0.109 | 0.913 | -0.200, 0.223 |
| **Education level** (ref: Primary school and below) |  |  |  |  |  |
| Junior high school | 0.118 | 0.116 | 1.017 | 0.309 | -0.110, 0.347 |
| Senior high school | 0.090 | 0.139 | 0.650 | 0.516 | -0.182, 0.363 |
| University or above | 0.144 | 0.215 | 0.670 | 0.503 | -0.278, 0.566 |
| **Medical insurance** (ref: Self-pay) |  |  |  |  |  |
| NBMI for urban employed | -0.910 | 0.306 | -2.979 | 0.003 | -1.510, -0.311 |
| NBMI for urban and rural residents | -0.708 | 0.318 | -2.228 | 0.026 | -1.331, -0.085 |
| **History of COPD** (ref: No) |  |  |  |  |  |
| Yes | 0.620 | 0.220 | 2.826 | 0.005 | 0.190, 1.051 |
| **History of cancer** (ref: No) |  |  |  |  |  |
| Yes | -2.620 | 6.007 | -0.436 | 0.663 | -14.401, 9.160 |
| **Family history of cancer** (ref: No) |  |  |  |  |  |
| Uncertain | 0.652 | 0.216 | 3.022 | 0.003 | 0.229, 1.076 |
| Yes | 0.300 | 0.276 | 1.086 | 0.277 | -0.242, 0.843 |
| **Consider LDCT as LCS method** (ref: No) |  |  |  |  |  |
| Yes | 0.047 | 0.093 | 0.509 | 0.611 | -0.135, 0.230 |
| **LDCT should be conducted once a year** (ref: No) |  |  |  |  |  |
| Yes | 0.085 | 0.089 | 0.960 | 0.337 | -0.089, 0.260 |
| **History of occupational exposure** (ref: No) |  |  |  |  |  |
| Yes | 0.301 | 0.111 | 2.718 | 0.007 | 0.084, 0.518 |
| **History of LCS** (ref: No) |  |  |  |  |  |
| Yes | 0.199 | 0.094 | 2.114 | 0.035 | 0.014, 0.383 |
| **Occupation** (ref: Worker) |  |  |  |  |  |
| Civil servant | 0.567 | 0.254 | 2.233 | 0.026 | 0.069, 1.065 |
| Businessperson | -0.024 | 0.182 | -0.132 | 0.895 | -0.381, 0.333 |
| Retired / Unemployed | -0.064 | 0.147 | -0.434 | 0.664 | -0.353, 0.225 |
| **Per capita monthly income** (ref: <$143) |  |  |  |  |  |
| $144–$428 | 0.303 | 0.172 | 1.763 | 0.078 | -0.034, 0.640 |
| $429–$713 | 0.654 | 0.187 | 3.502 | <0.001 | 0.288, 1.020 |
| ≥$714 | 0.617 | 0.206 | 2.998 | 0.003 | 0.213, 1.021 |
| **Smoking status** (ref: No) |  |  |  |  |  |
| Currently smoking | 0.208 | 0.155 | 1.339 | 0.181 | -0.096, 0.512 |
| Quit smoking | 0.321 | 0.179 | 1.796 | 0.073 | -0.030, 0.671 |

## Appendix 3 Table 4. Multiple Linear Regression Analysis of Factors Associated with Perceived Self-Efficacy for Lung Cancer Screening (HC3 analysis)

| **Variable** | **B** | **Robust SE** | **t** | **p** | **95% CI** |
| --- | --- | --- | --- | --- | --- |
| Intercept | 25.878 | 1.670 | 15.491 | <0.001 | 22.601, 29.154 |
| **Sex** (ref: Male) |  |  |  |  |  |
| Female | -0.591 | 0.357 | -1.655 | 0.098 | -1.290, 0.109 |
| Age | -0.029 | 0.016 | -1.775 | 0.076 | -0.060, 0.003 |
| BMI | 0.002 | 0.023 | 0.085 | 0.933 | -0.044, 0.047 |
| **Religion** (ref: No) |  |  |  |  |  |
| Yes | 0.202 | 0.238 | 0.848 | 0.396 | -0.264, 0.668 |
| **Residential area** (ref: Urban) |  |  |  |  |  |
| Rural | 1.185 | 0.257 | 4.616 | <0.001 | 0.681, 1.688 |
| **Education level** (ref: Primary school and below) |  |  |  |  |  |
| Junior high school | 0.759 | 0.285 | 2.664 | 0.008 | 0.200, 1.318 |
| Senior high school | 0.866 | 0.342 | 2.531 | 0.011 | 0.195, 1.537 |
| University or above | 1.261 | 0.579 | 2.177 | 0.030 | 0.125, 2.397 |
| **Medical insurance** (ref: Self-pay) |  |  |  |  |  |
| NBMI for urban employed | -1.394 | 0.777 | -1.794 | 0.073 | -2.919, 0.130 |
| NBMI for urban and rural residents | -1.976 | 0.797 | -2.481 | 0.013 | -3.539, -0.414 |
| **History of COPD** (ref: No) |  |  |  |  |  |
| Yes | -1.199 | 0.520 | -2.303 | 0.021 | -2.219, -0.178 |
| **History of cancer** (ref: No) |  |  |  |  |  |
| Yes | 1.680 | 0.673 | 2.497 | 0.013 | 0.360, 3.001 |
| **Family history of cancer** (ref: No) |  |  |  |  |  |
| Uncertain | -0.853 | 0.504 | -1.695 | 0.090 | -1.841, 0.134 |
| Yes | -0.137 | 0.842 | -0.162 | 0.871 | -1.788, 1.515 |
| **Consider LDCT as LCS method** (ref: No) |  |  |  |  |  |
| Yes | 1.719 | 0.216 | 7.948 | <0.001 | 1.295, 2.143 |
| **LDCT should be conducted once a year** (ref: No) |  |  |  |  |  |
| Yes | 1.632 | 0.206 | 7.915 | <0.001 | 1.227, 2.036 |
| **History of occupational exposure** (ref: No) |  |  |  |  |  |
| Yes | -0.641 | 0.253 | -2.533 | 0.011 | -1.138, -0.145 |
| **History of LCS** (ref: No) |  |  |  |  |  |
| Yes | 1.962 | 0.230 | 8.515 | <0.001 | 1.510, 2.414 |
| **Occupation** (ref: Worker) |  |  |  |  |  |
| Civil servant | -0.601 | 0.638 | -0.942 | 0.347 | -1.853, 0.651 |
| Businessperson | 0.016 | 0.441 | 0.037 | 0.970 | -0.848, 0.881 |
| Retired / Unemployed | -0.685 | 0.383 | -1.786 | 0.074 | -1.436, 0.067 |
| **Per capita monthly income** (ref: <$143) |  |  |  |  |  |
| $144–$428 | 0.830 | 0.449 | 1.847 | 0.065 | -0.051, 1.711 |
| $429–$713 | 1.456 | 0.493 | 2.952 | 0.003 | 0.489, 2.424 |
| ≥$714 | 2.430 | 0.536 | 4.534 | <0.001 | 1.379, 3.481 |
| **Smoking status** (ref: No) |  |  |  |  |  |
| Currently smoking | -0.042 | 0.405 | -0.104 | 0.917 | -0.836, 0.752 |
| Quit smoking | -0.488 | 0.429 | -1.137 | 0.256 | -1.330, 0.354 |

## Appendix 3 Table 5. Multiple Linear Regression Analysis of Factors Associated with Total LCS Health Belief Score (HC3 analysis)

| **Variable** | **B** | **Robust SE** | **t** | **p** | **95% CI** |
| --- | --- | --- | --- | --- | --- |
| Intercept | 85.418 | 3.247 | 26.308 | <0.001 | 79.051, 91.786 |
| **Sex** (ref: Male) |  |  |  |  |  |
| Female | -1.627 | 0.704 | -2.311 | 0.021 | -3.008, -0.246 |
| Age | 0.057 | 0.029 | 1.939 | 0.053 | -0.001, 0.115 |
| BMI | 0.018 | 0.051 | 0.360 | 0.719 | -0.081, 0.118 |
| **Religion** (ref: No) |  |  |  |  |  |
| Yes | 0.917 | 0.454 | 2.021 | 0.043 | 0.027, 1.807 |
| **Residential area** (ref: Urban) |  |  |  |  |  |
| Rural | 3.276 | 0.495 | 6.622 | <0.001 | 2.306, 4.246 |
| **Education level** (ref: Primary school and below) |  |  |  |  |  |
| Junior high school | 0.698 | 0.506 | 1.380 | 0.168 | -0.294, 1.691 |
| Senior high school | 1.120 | 0.636 | 1.761 | 0.078 | -0.127, 2.366 |
| University or above | 1.187 | 1.102 | 1.078 | 0.281 | -0.973, 3.348 |
| **Medical insurance** (ref: Self-pay) |  |  |  |  |  |
| NBMI for urban employed | -2.894 | 1.648 | -1.757 | 0.079 | -6.125, 0.337 |
| NBMI for urban and rural residents | -4.122 | 1.741 | -2.368 | 0.018 | -7.536, -0.709 |
| **History of COPD** (ref: No) |  |  |  |  |  |
| Yes | -4.820 | 0.831 | -5.797 | <0.001 | -6.451, -3.189 |
| **History of cancer** (ref: No) |  |  |  |  |  |
| Yes | 5.089 | 128.006 | 0.040 | 0.968 | -245.955, 256.133 |
| **Family history of cancer** (ref: No) |  |  |  |  |  |
| Uncertain | -1.212 | 0.919 | -1.319 | 0.187 | -3.014, 0.590 |
| Yes | 0.632 | 1.523 | 0.415 | 0.678 | -2.355, 3.619 |
| **Consider LDCT as LCS method** (ref: No) |  |  |  |  |  |
| Yes | 4.912 | 0.416 | 11.818 | <0.001 | 4.097, 5.728 |
| **LDCT should be conducted once a year** (ref: No) |  |  |  |  |  |
| Yes | 4.640 | 0.390 | 11.883 | <0.001 | 3.874, 5.405 |
| **History of occupational exposure** (ref: No) |  |  |  |  |  |
| Yes | -0.911 | 0.493 | -1.845 | 0.065 | -1.878, 0.057 |
| **History of LCS** (ref: No) |  |  |  |  |  |
| Yes | 5.717 | 0.438 | 13.047 | <0.001 | 4.857, 6.576 |
| **Occupation** (ref: Worker) |  |  |  |  |  |
| Civil servant | -1.640 | 1.336 | -1.228 | 0.220 | -4.260, 0.980 |
| Businessperson | -0.914 | 0.829 | -1.102 | 0.270 | -2.539, 0.712 |
| Retired / Unemployed | -0.309 | 0.690 | -0.448 | 0.654 | -1.663, 1.045 |
| **Per capita monthly income** (ref: <$143) |  |  |  |  |  |
| $144–$428 | 1.917 | 0.846 | 2.267 | 0.024 | 0.258, 3.576 |
| $429–$713 | 3.142 | 0.916 | 3.431 | 0.001 | 1.346, 4.937 |
| ≥$714 | 3.586 | 1.025 | 3.499 | <0.001 | 1.576, 5.596 |
| **Smoking status** (ref: No) |  |  |  |  |  |
| Currently smoking | -1.722 | 0.772 | -2.230 | 0.026 | -3.237, -0.208 |
| Quit smoking | -1.802 | 0.867 | -2.079 | 0.038 | -3.502, -0.102 |
